# Association between the triglyceride-glucose index and 1-year major adverse cardiovascular events in patients with coronary heart disease and hypertension

**DOI:** 10.1186/s12933-023-02018-9

**Published:** 2023-11-08

**Authors:** Shiyi Tao, Lintong Yu, Jun Li, Li Huang, Xuanchun Huang, Wenjie Zhang, Zicong Xie, Yuqing Tan, Deshuang Yang

**Affiliations:** 1 Department of Cardiology, Guang’anmen Hospital, China Academy of Chinese Medical Sciences, Beijing, China; 2 Graduate School, Beijing University of Chinese Medicine, Beijing, China; 3 Department of Integrative Cardiology, China-Japan Friendship Hospital, Beijing, China

**Keywords:** Triglyceride-glucose index, Major adverse cardiovascular events, Coronary Heart Disease, Hypertension, Insulin resistance

## Abstract

**Background:**

The triglyceride-glucose (TyG) index has been proposed as a potential predictor of adverse prognosis of coronary heart disease (CHD). However, its prognostic value in patients with CHD and hypertension remains unclear. This study aimed to evaluate the association between the TyG index and the 1-year risk of major adverse cardiovascular events (MACEs) in patients with CHD and hypertension.

**Methods:**

The data for the study were taken from the Hospital Information System database in China-Japan Friendship Hospital which contained over 10,000 cardiovascular admissions from 2019 to 2022. The Boruta algorithm was performed for feature selection. The study used univariable analysis, multivariable logistic regression analysis, and restricted cubic spline (RCS) regression to evaluate the association between the TyG index and the 1-year risk of MACEs in patients with CHD and hypertension.

**Results:**

After applying inclusion and exclusion criteria, a total of 810 patients with CHD and hypertension were included in the study with a median TyG index of 8.85 (8.48, 9.18). Using the lowest TyG index quartile as the reference, the fully adjusted ORs (95% CIs) for 1-year MACEs for TyG index Q2, Q3, and Q4 were 1.001 (0.986 ~ 1.016), 1.047 (1.032 ~ 1.062), and 1.760 (1.268 ~ 2.444), respectively. After adjusting for all confounders, we found that those with the highest TyG index had a 47.0% increased risk of MACEs over the 1-year follow-up (OR 1.470, 95% *CI* 1.071 ~ 2.018). The results in the subgroup analysis were similar to the main analyses. RCS model suggested that the TyG index was nonlinearly associated with the 1-year risk of MACEs (*P* for nonlinear < 0.001).

**Conclusion:**

This study shows that the elevated TyG index is a potential marker of adverse prognosis among patients with CHD and hypertension and informs the development of clinical decisions to improve outcomes.

**Supplementary Information:**

The online version contains supplementary material available at 10.1186/s12933-023-02018-9.

## Introduction

Coronary heart disease (CHD) is the leading cause of human death and loss of healthy life, ranking first in the global disease burden [1]. The prevalence of CHD is on the rise in China, with an estimated 330 million patients suffering from CHD [2]. Hypertension is a leading cause of long-term major adverse cardiovascular events (MACEs). Early detection and prompt management of cardiovascular disease (CVD) risk in hypertensive patients is imperative to guide clinicians and decrease CVD burdens worldwide [3, 4].

Insulin resistance (IR) has long been recognized as a risk factor for both micro- and macroangiopathies [5]. The triglyceride-glucose (TyG) index has been regarded as a reliable and surrogate measure of IR, and it has been reported to be more diagnostic and predictive of diabetes [6–8]. Previous research has proven that the TyG index is significantly correlated with the development of cardiovascular disease [9], the risk of myocardial infarction [10], in-stent stenosis [11], the severity of coronary artery disease [12], etc. Furthermore, disturbances in glycometabolism are common in patients with hypertension [13], and studies have suggested a link between TyG and hypertension prognosis [14]. However, the pathophysiology of hypertensive patients combined with CHD is complex and the association between the TyG index and the 1-year risk of MACEs in patients with CHD and hypertension remains unclear.

This study aimed to explore the association between the TyG index and the 1-year risk of MACEs in patients with CHD and hypertension based on accessible and detailed clinical information. The results of this study may help develop new strategies to improve patient prognosis in this population and provide important new insights into the function of TyG in predicting patient outcomes.

## Materials and methods

### Study design and participants

We retrospectively assessed the admission data of consecutive hypertensive patients from the Hospital Information System database in China-Japan Friendship Hospital between September 2019 and March 2022. All eligible participants ranged from 18 to 80 years fulfilled the diagnostic criteria for CHD and hypertension and MACEs were assessed with 1-year follow-up. Among the 5684 candidates, 4874 participants were excluded based on the study exclusion criteria, including patients (1) not first admission (*n* = 4137), (2) did not meet the age requirement (*n* = 54), (3) with serious diseases (*n* = 241), (4) with secondary hypertension (*n* = 96), (5) with > 30% missing baseline-related data (*n* = 61), and (6) with missing data (fasting blood glucose, triglycerides, percutaneous coronary angiography) (*n* = 285). Finally, a total of 810 participants were enrolled in this study. Included patients were divided into MACEs group (*n* = 379) and non-MACEs group (*n* = 431) according to the occurrence of MACEs within one year.

CHD was defined as having at least one of the following conditions [15]: (1) percutaneous coronary angiography or computed tomographic angiography examination showed that at least one coronary artery trunk or primary branch had ≥ 50% stenosis; (2) typical exertional angina symptoms with positive stress test (electrocardiogram stress test, stress echocardiography or nuclide myocardial stress imaging); (3) previously diagnosed MI or unstable angina pectoris. The ESH hypertension Guidelines were used to define the initial diagnosis of hypertension defined as systolic blood pressure (SBP) was greater than 140 mmHg or their diastolic blood pressure (DBP) was no less than 90 mmHg [16].

### Data collection and definitions

The data were obtained and refined from previous inpatient and outpatient medical records. Following enrollment, baseline data on each patient was collected by trained investigations using a standardized questionnaire, including demographic characteristics, clinical history, laboratory indicators, echocardiography and peripheral arterial disease features, and number of coronary lesions.

Demographic characteristics included weight, height [to calculate body mass index (BMI)], baseline blood pressure, age, gender, heart rate (HR), smoking history and drinking history. Clinical history included established history of CVDs, diabetes, old myocardial infarction (OMI), chronic kidney disease (CKD). Laboratory indicators of blood samples were fasting venous blood collected by professional medical staff from all participants in the early morning, including total cholesterol (TC), triglyceride (TG), low density lipoprotein cholesterol (LDL-C), high density lipoprotein cholesterol (HDL-C), homocysteine (HCY), hypersensitive C-reactive protein (Hs-CRP), serum creatinine (Scr), fasting blood glucose (FBG) and glycated hemoglobin A1c (HbA1c). The units of FBG and TG were first converted from mmol/L to mg/dL and the TyG index was calculated as Ln [fasting TG (mg/dL) × FBG (mg/dL)/2]. Echocardiography features included left atrial diameter (LAD), left ventricular end-diastolic diameter (LVDd), interventricular septal thickness (IVST), left ventricular posterior wall thickness (PWT), and left ventricular ejection fraction (LVEF). Peripheral arterial disease indicators included brachial-ankle pulse wave velocity (baPWV), ankle-brachial index (ABI), and brachial artery flow-mediated vasodilatation (FMD) value.

### Feature selection

We utilized the Boruta algorithm to determine the most critical features related to 1-year MACEs and to construct the radiomics signatures. The Boruta algorithm is an extension of the random forest algorithm and involves the creation of “shadow features” by shuffling the real features. In each iteration of the algorithm, the Z-value of each feature is calculated based on its importance in the random forest model, and the maximum Z-value of the shadow features is recorded. A real feature is considered important if its Z-value is greater than the maximum Z-value of the shadow features; otherwise, it was eliminated. The default parameters used for the Boruta algorithm are “*P* value = 0.01” and “maxRuns = 100”, which represent the level of significance for feature selection and the maximum number of iterations for the algorithm, respectively [17].

### Follow-Up and endpoints

Clinical follow-up was carried out by skilled clinicians in outpatient or telephone contact at the time points of one year, and standard computerized case report forms were filled out. The endpoint events were independently categorized by three cardiovascular specialists who were not aware of the baseline information. When there were disagreements regarding event identification, the three experts came to a decision together after talking.

The primary endpoint of this clinical trial was defined as a compound endpoint of the first occurrence of total MACEs within one-year follow up. The total MACEs was defined as follows: (1) cardiac death, including fatal events caused by coronary artery disease or myocardial infarction; (2) non-fatal myocardial infarction, referring to myocardial necrosis but no death, accompanied by ischemia symptoms, abnormal myocardial markers, ST segment changes or pathological Q wave changes; (3) unplanned revascularization, which means that the patient underwent revascularization again due to unexpected internal cardiac causes; (4) in-stent restenosis, which was defined as 50% or more of the target vessel stenosis within 5 mm from the edge of the stent or both ends of the stent after percutaneous coronary intervention as shown by coronary angiography; (5) stroke, including cerebral infarction, cerebral hemorrhage and subarachnoid hemorrhage; and (6) unplanned rehospitalization for cardiac causes (unstable angina pectoris, acute exacerbation of chronic heart failure, etc.).

### Statistical analysis

All statistical analyses were performed using IBM-SPSS (version 26.0, Chicago, IL, USA) and R (version 4.1.2, Vienna, Austria). The subjects were classified into different groups according to the occurrence of MACEs within 1-year follow-up. Continuous variables presented as median and interquartile range were tested using the Wilcoxon rank sum test. Categorical variables were summarized as percentage-based figures and compared by the Chi-Square test. The cumulative incidence of 1-year MACEs in groups were analyzed using the Kaplan-Meier curve.

To evaluate the relationship between the TyG index and the risk of 1-year MACEs, univariable and multivariable logistic regression analyses were conducted. Model 1 contained only the TyG index without any other adjustments for confounding factors. In Model 2, gender and age were modified. Model 3 was a completely adjusted model that took feature selection results and clinical experience adjustments into account. Additionally, restricted cubic spline (RCS) regression was used to assess any potential nonlinear relationships between the TyG index and the risk of 1-year MACEs in patients with CHD and hypertension. We also performed subgroup analysis based on age (< 65 years or ≥ 65 years), gender (male or female), diabetes (yes or no), family history of CVDs (yes or no) and multivessel disease (yes or no) to determine whether the correlation between TyG index and 1-year MACEs in different subgroups was different, and the *P* value of the interaction was calculated. A two-sides *P* value of less than 0.05 was considered to indicate statistical significance.

## Results

### Baseline characteristics

**Fig. 1:**
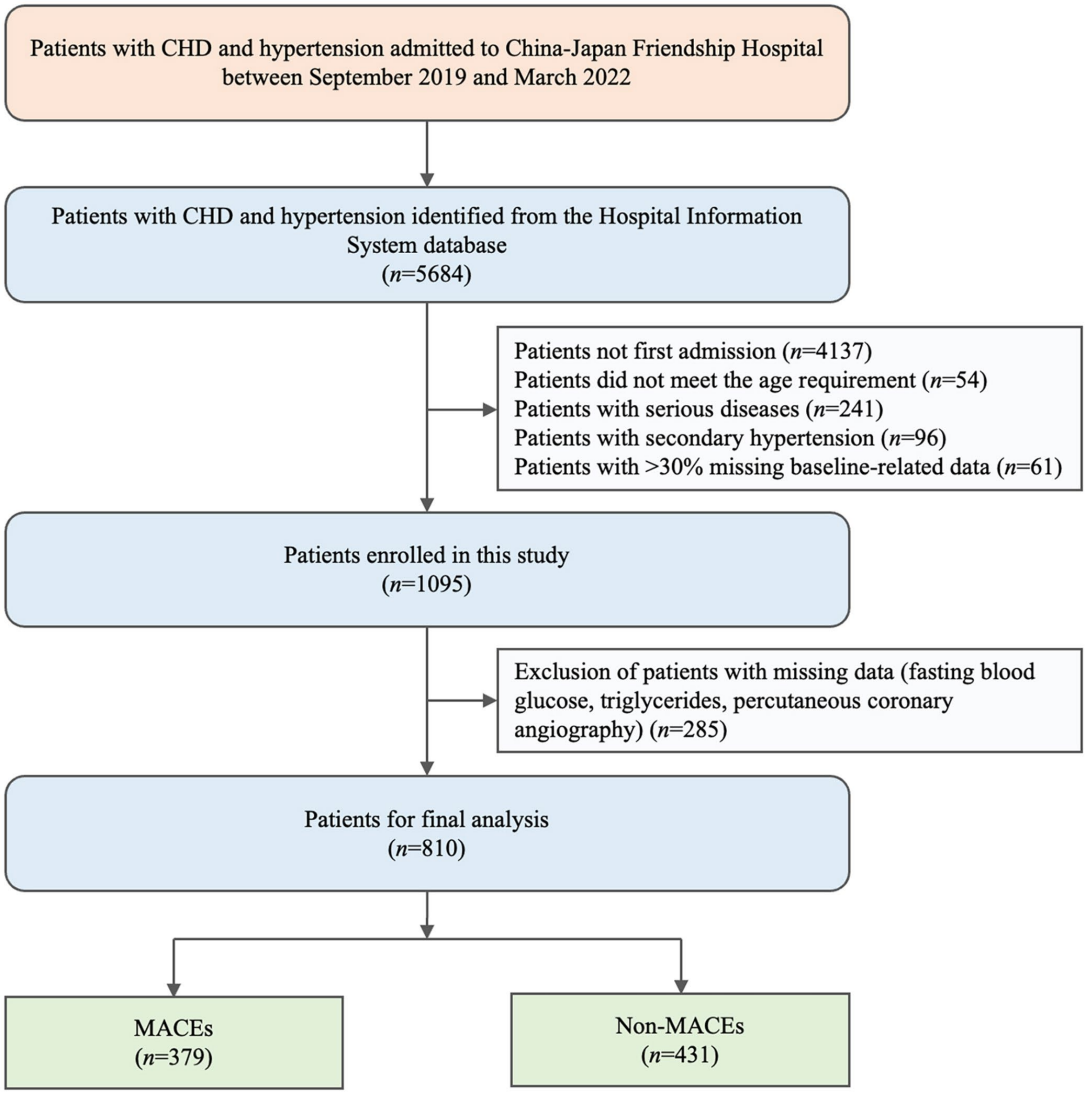
Flowchart of the detailed selection process. CHD, coronary heart disease; MACE, major adverse cardiovascular event

The inclusion and exclusion criteria led to the inclusion of 810 patients with CHD and hypertension from the Hospital Information System database in the study (Fig. 1). The median TyG index was 8.85 (8.48, 9.18). Of the 810 patients with CHD and hypertension who were hospitalized, 379 (46.79%) suffered from MACEs within one year while 431 others did not.

The variations in baseline characteristics are summarized in Table 1 and Additional file 1: Table S1. Age, BMI, SBP, HR, TG, HCY, and Scr were greater both in patients with higher baseline TyG index and who suffered from MACEs within one year, and they also had increased risks for dysglycemia and were more likely to have worse cardiac and peripheral artery conditions (*P* < 0.001). MACEs group had a higher proportion of patients with a family history of CVDs, and patients tended to be diagnosed with CKD and OMI (*P* < 0.001). Besides, patients with one- and two-vessel disease had a higher risk of MACEs (*P* < 0.05). However, no significant difference was observed between patients with multivessel disease in different MACEs groups (*P* = 0.210) and TyG quartiles groups (*P* = 0.375), possibly due to the small sample size. Collinearity diagnostics implied that no potentially significant collinearity was found among these variables (Additional file 1: Table S2).

**Table 1:** The main baseline characteristics for eligible patients divided by MACEs-related situation

Indicators	Overall	MACEs	Non-MACEs	*P* value
N	810	379	431	
Male (n, %)	571 (70.49)	277 (73.09)	294 (68.21)	0.129
Age (years)	66 (57, 74)	69 (61, 78)	63 (55, 70)	< 0.001
BMI (kg/m^2^)	24.97 (23.38, 27.22)	25.95 (24.27, 28.23)	24 (22.84, 25.96)	< 0.001
SBP (mmHg)	136 (123, 149)	139 (126, 156)	132 (122, 145)	< 0.001
DBP (mmHg)	79 (70, 86)	80 (70, 87)	79 (70, 85)	0.888
HR (bpm)	72 (68, 80)	76 (70, 82)	70 (66, 76)	< 0.001
Smoking (n, %)	358 (44.2)	168(44.33)	190 (44.08)	0.944
Drinking (n, %)	197 (24.32)	99 (26.12)	98 (22.74)	0.263
Case history (n, (%)				
Diabetes	403 (49.75)	203 (47.1)	200 (52.77)	0.107
CKD ^a^	114 (14.07)	85 (22.43)	29 (6.73)	< 0.001
OMI	151 (18.64)	110 (29.02)	41 (9.51)	< 0.001
Family history of CVDs	258 (31.85)	157 (41.42)	101 (23.43)	< 0.001
Number of coronary lesions (n, %)				
One-vessel disease	629 (77.65)	279 (73.61)	350 (81.21)	0.010
Two-vessel disease	146 (18.02)	80 (21.11)	66 (15.31)	0.032
Multi-vessel disease	35 (4.32)	20 (5.28)	15 (3.48)	0.210
Cardiovascular medications (n, %)				
Anti-platelet	809 (99.88)	379 (100)	430 (99.77)	0.348
Statins	796 (98.27)	373 (98.42)	423 (98.14)	0.766
ACEI/ARB	552 (68.15)	246 (64.91)	306 (71)	0.063
*β*-blockers	613 (75.68)	298 (78.63)	315 (73.09)	0.067
CCB	344 (42.47)	148 (39.05)	196 (45.48)	0.065
Nitrates	251 (30.99)	113 (29.82)	138 (32.02)	0.499
Laboratory variables				
TC (mmol/L)	3.83 (3.2, 4.71)	3.76 (3.18, 4.98)	3.83 (3.24, 4.57)	0.290
TG (mmol/L)	1.44 (1.06, 1.94)	1.65 (1.24, 2.04)	1.26 (1.03, 1.74)	< 0.001
LDL-C (mmol/L)	2.28 (1.81, 2.86)	2.24 (1.71, 2.91)	2.26 (1.87, 2.78)	0.487
HDL-C (mmol/L)	0.99 (0.83, 1.15)	0.95 (0.76, 1.1)	0.99 (0.87, 1.18)	0.002
HCY (*µ*mol/L)	14.11 (11.38, 17.97)	16.45(12.99, 21.75)	12.43 (10.7, 15.48)	< 0.001
Hs-CRP (mg/L)	2.08 (0.97, 4.58)	3.45 (1.73, 9.13)	1.44 (0.71, 2.47)	< 0.001
Scr (*µ*mol/L)	75.8 (62.9, 90.3)	79.8 (65.48, 99.08)	72.5 (60.7, 84.3)	< 0.001
FBG (mmol/L)	5.91 (5.13, 6.9)	6.23 (5.27, 7.51)	5.61 (5, 6.62)	< 0.001
HbA1c (%)	6.2 (5.8, 7.1)	6.5 (5.9, 7.73)	6.1 (5.7, 6.8)	< 0.001
TyG index	8.85 (8.48, 9.18)	9.07 (8.72, 9.29)	8.72 (8.41, 8.98)	< 0.001
PAD indicators				
baPWV (m/s)	17.2 (15.38, 22.1)	22.66 (20.63, 25.09)	15.48 (14.68, 16.21)	< 0.001
ABI	1.08 (0.92, 1.17)	0.92 (0.8, 1)	1.16 (1.12, 1.2)	< 0.001
FMD (%)	6.8 (6, 8)	6 (5.6, 6.4)	7.6 (7, 9)	< 0.001
Echocardiography				
LAD (mm)	38 (36, 40)	40 (37, 43)	37 (35, 38)	< 0.001
LVDd (mm)	51 (48, 54)	53 (50, 56)	49 (46, 52)	< 0.001
IVST (mm)	10 (9, 11)	11 (10, 12)	10 (9, 10)	< 0.001
PWT (mm)	9 (8, 10)	10 (9, 10)	9 (8, 9)	< 0.001
LVEF (%)	65 (60, 69)	61.5 (55, 67)	67 (63, 71)	< 0.001

MACE, major adverse cardiovascular event; BMI, body mass index; SBP, systolic blood pressure; DBP, diastolic blood pressure; HR, heart rate; CKD, chronic kidney disease; OMI, old myocardial infarction; CVD, cardiovascular disease; ACEI, angiotensin converting enzyme inhibitor; ARB, angiotensin receptor blocker; CCB, calcium channel blockers; TC, total cholesterol; TG, triglyceride; LDL-C, low-density lipoprotein cholesterol; HDL-C, high-density lipoprotein cholesterol; HCY, homocysteine; Hs-CRP, hypersensitive C-reactive protein; Scr, serum creatinine; FBG, fasting blood glucose; HbA1c, glycosylated hemoglobin; TyG, triglyceride-glucose; PAD, peripheral artery disease; baPWV, brachial-ankle pulse wave velocity; ABI, ankle-brachial index; FMD, brachial artery flow-mediated vasodilatation; LAD, left atrial diameter; LVDd, left ventricular end-diastolic diameter; IVST, interventricular septal thickness; PWT, left ventricular posterior wall thickness; LVEF, left ventricular ejection fraction

^a^ Defined as eGFR < 60 ml/min/1.73 m^2^ on the basis of The KDIGO CKD Clinical Guideline

### Feature selection

**Fig. 2:**
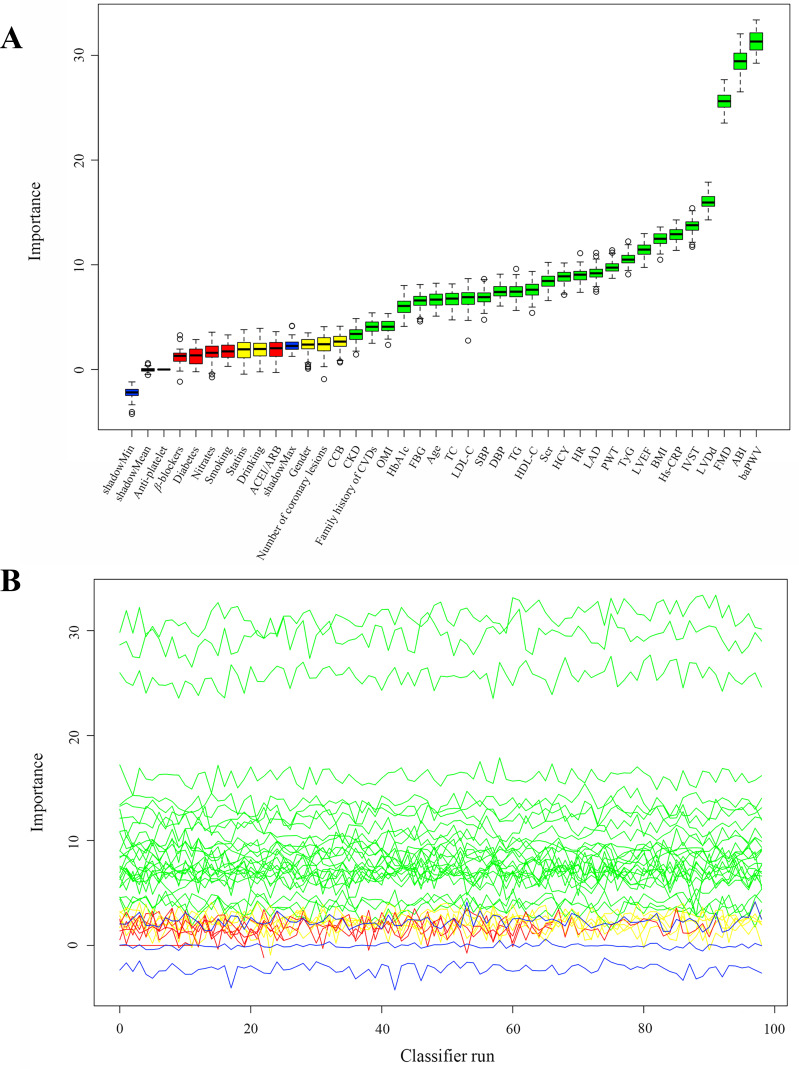
Feature selection for the relationship between various TyG indices and the risk of 1-year MACEs analyzed by the Boruta algorithm. (**A**). The process of feature selection. (**B**). The value evolution of Z-score in the screening process. The horizontal axis shows the name of each variable and the number of iterations for the algorithm in Fig. 2-A and -B, respectively. While the vertical axis represents the Z-value of each variable. The green boxes and lines represent confirmed variables, the yellow ones represent tentative attributes, and the red ones represent rejected variables in the model calculation. TyG, triglyceride-glucose; BMI, body mass index; SBP, systolic blood pressure; DBP, diastolic blood pressure; HR, heart rate; CKD, chronic kidney disease; OMI, old myocardial infarction; CVD, cardiovascular disease; ACEI, angiotensin converting enzyme inhibitor; ARB, angiotensin receptor blocker; CCB, calcium channel blockers; TC, total cholesterol; TG, triglyceride; LDL-C, low-density lipoprotein cholesterol; HDL-C, high-density lipoprotein cholesterol; HCY, homocysteine; Hs-CRP, hypersensitive C-reactive protein; Scr, serum creatinine; FBG, fasting blood glucose; HbA1c, glycosylated hemoglobin; baPWV, brachial-ankle pulse wave velocity; ABI, ankle-brachial index; FMD, brachial artery flow-mediated vasodilatation; LAD, left atrial diameter; LVDd, left ventricular end-diastolic diameter; IVST, interventricular septal thickness; PWT, left ventricular posterior wall thickness; LVEF, left ventricular ejection fraction

Twenty-six variables that were the most associated with the risk of 1-year MACEs were confirmed important using the Boruta method (Fig. 2). Although several important characteristics, such as gender, diabetes and medication situation, such as anti-platelet and statins use, were disregarded because of the low Z-value in comparison to the shadow feature, they were nonetheless included in the analysis based on prior research and clinical experience. Factors were chosen for the final complete adjustment model when in the Boruta analysis, their Z-scores were higher than the shadow features or when added to the model, they had the largest matched effect (odds ratio or hazard ratio) among a group of biomarkers (max, mean and min) or they were based on previous findings and clinical constraints.

### TyG index and 1-year MACEs relationship

**Fig. 3:**
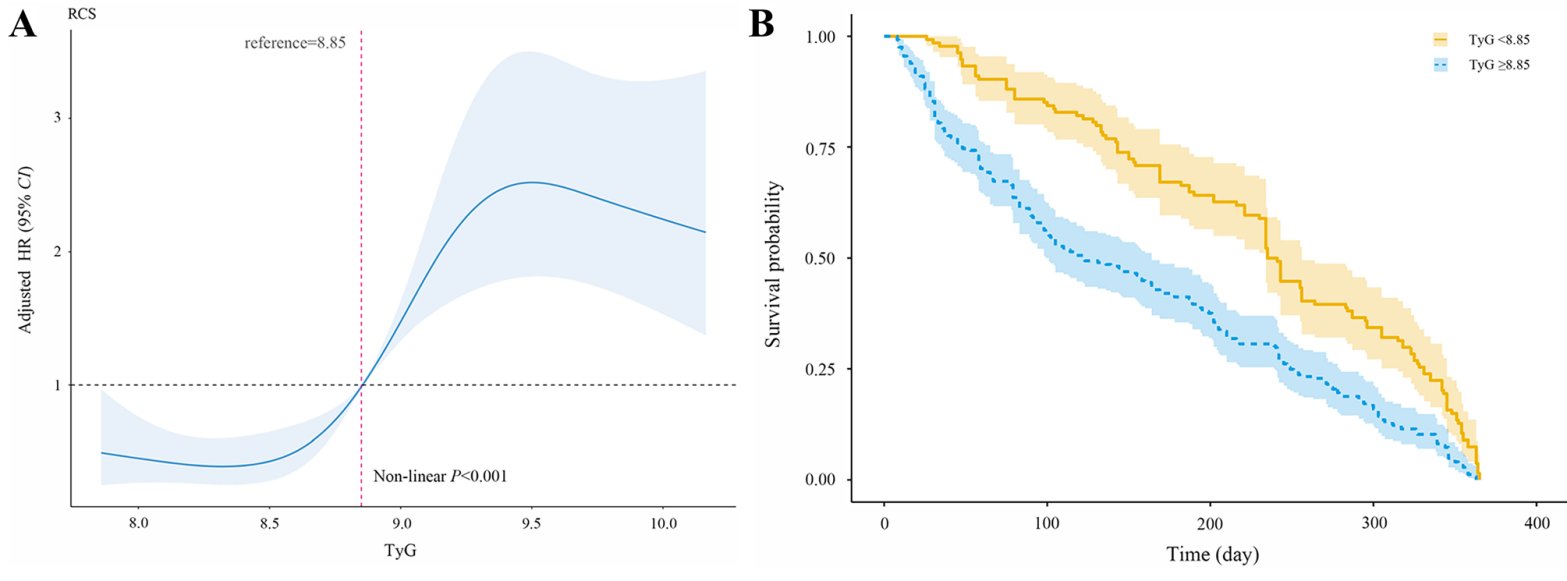
Analysis of the relationship between the TyG index and the risk of 1-year MACEs. (**A**) Multivariable RCS regression showed the nonlinear association between the TyG index and the risk of 1-year MACEs after full adjustment. The cut-off value of TyG index in predicting MACEs was 8.85; (**B**) Kaplan-Meier analysis results illustrated the cumulative incidence of 1-year risk of MACEs in patients with both CHD and hypertension in various groups divided by the cut-off value of TyG index. TyG triglyceride-glucose; RCS, restricted cubic spline

According to the follow-up, 379 had MACEs within one year out of 810 patients (46.79%). The TyG index was found to have a nonlinear relationship with the probability of the risk of 1-year MACEs according to the multivariable RCS model. The cut-off value for the TyG index was calculated automatically during the analysis using RCS regression method. When the TyG index was 8.85, the 1-year risk of MACEs was differentiated, and the hazard ratio (HR) value of the TyG index was near 1(Fig. 3A). The Kaplan-Meier analysis plot showed a significant difference among various groups divided by the cut-off value of TyG index (*P* < 0.001) (Fig. 3B).

**Fig. 4:**
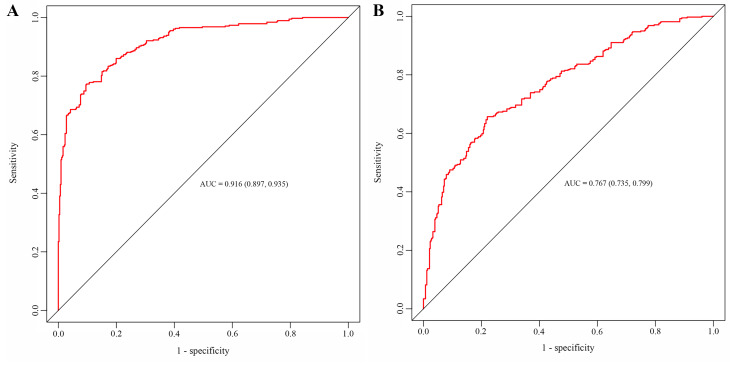
Performance evaluation of Model 3 and the conventional model. The area under the curve of (**A**) Model 3 and (**B**) conventional model was analyzed by the receiver operating characteristic curve analysis

We defined four categories of patients based on the quartiles of the TyG index: Q1 (TyG ≤ 8.48), Q2 (8.48 < TyG ≤ 8.85), Q3 (8.85 < TyG ≤ 9.18), and Q4 (TyG > 9.18). The results of multivariable logistic regression (Table 2, Model 3) showed that the TyG index increased the probability of the risk of 1-year MACEs (OR 1.470, 95% *CI* 1.071 ~ 2.018) after adjusting for all impact factors identified by Boruta analysis and clinical experience. The trend from Q 1 to Q 4 was statistically significant (Table 2, P for trend 0.019). Compared to the lowest TyG index in Q1 (Table 2, P for trend 0.038), the OR for the incidence of 1-year MACEs decreased in Q2 (OR 1.001, 95% *CI* 0.986 ~ 1.016) and Q3 (OR 1.047, 95% *CI* 1.032 ~ 1.062); however, it climbed in Q3 (OR 1.760, 95% *CI* 1.268 ~ 2.444). Furthermore, the receiver operating characteristic curve analysis showed that model 3 had a better performance with an area under the curve (AUC) of 0.916 (0.897 ~ 0.935) compared with the conventional model [AUC 0.767 (0.735 ~ 0.799)] (Fig. 4).

**Table 2:** The association between various TyG index groups and 1-year MACEs

	Model 1	Model 2	Model 3
TyG index	1.350 (0.992 ~ 1.836)	1.409 (1.023 ~ 1.941)	1.470 (1.071 ~ 2.018)
TyG			
Q1	1 (reference)	1 (reference)	1 (reference)
Q2	0.746 (0.498 ~ 1.120)	0.896 (0.790 ~ 1.017)	1.001 (0.986 ~ 1.016)
Q3	1.015 (1.006 ~ 1.024)	1.046 (1.031 ~ 1.016)	1.047 (1.032 ~ 1.062)
Q4	1.727 (1.246 ~ 2.393)	1.747 (1.250 ~ 2.440)	1.760 (1.268 ~ 2.444)
*P* for trend	0.038	0.044	0.019
Model 1	Unadjusted		
Model 2	Adjusted for age and gender		
Model 3	Adjusted for adjusting for all impact factors identified by Boruta analysis and clinical experience		

TyG, triglyceride-glucose; Q1, quartile 1; Q2, quartile 2; Q3, quartile 3; Q4, quartile 4

### Subgroup analysis

The association between the TyG index and 1-year MACEs was examined in the subgroups analysis according to age (< 65 years or ≥ 65 years), gender (male or female), diabetes (yes or no), family history of CVDs (yes or no) and multivessel disease (yes or no). As shown in Table 3, gender was found to interact with the relationship between the TyG index and MACEs among one year (*P* = 0.042). Elderly patients (≥ 65 years), patients with family history of CVDs and patients with or without multivessel disease continued to show a similar association between the TyG index and 1-year risk of MACEs.

The subgroup analysis was almost consistent with the major findings. This study showed that there was no significant association of the TyG index with 1-year MACEs in patients younger than 65 years of age or without diabetes (*P* > 0.05).

**Table 3:** The subgroup analysis results of the multivariable-adjusted ORs for the association between the TyG index and 1-year MACEs

	Case	Q1	Q2	Q3	Q4	*P* for trend	*P* for interaction
Age							
< 65	362	1 (reference)	1.040 (0.518 ~ 2.091)	1.358 (0.738 ~ 2.490)	1.680 (0.976 ~ 3.623)	0.052	0.067
≥ 65	448	1 (reference)	0.810 (0.482 ~ 1.359)	2.349 (0.482 ~ 5.359)	2.815 (1.225 ~ 5.484)	0.015	
Gender							
Male	571	1 (reference)	0.900 (0.550 ~ 1.473)	1.795 (0.757 ~ 3.447)	2.345 (1.077 ~ 4.389)	0.021	0.042
Female	239	1 (reference)	0.955 (0.430 ~ 2.120)	1.525 (0.527 ~ 3.135)	2.285 (1.272 ~ 3.959)	0.026	
Diabetes							
Yes	403	1 (reference)	0.589 (0.311 ~ 1.115)	1.972 (1.048 ~ 3.711)	2.393 (1.331 ~ 4.300)	0.019	0.395
No	407	1 (reference)	1.094 (0.634 ~ 1.886)	1.107 (0.805 ~ 2.291)	1.521 (0.705 ~ 2.841)	0.071	
Family history of CVDs							
Yes	258	1 (reference)	1.042 (0.510 ~ 2.129)	1.915 (0.921 ~ 3.980)	3.126 (1.284 ~ 6.207)	0.005	0.362
No	552	1 (reference)	0.731 (0.432 ~ 1.238)	2.465 (1.079 ~ 4.773)	2.470 (1.121 ~ 4.676)	0.018	
Multi-vessel disease							
Yes	35	1 (reference)	1.184 (0.716 ~ 2.782)	2.625 (0.450 ~ 5.311)	3.294 (1.119 ~ 5.952)	0.003	0.205
No	775	1 (reference)	0.909 (0.299 ~ 1.381)	2.741 (1.813 ~ 4.143)	3.806 (2.481 ~ 5.840)	0.001	

TyG, triglyceride-glucose; CVD, cardiovascular disease; Q1, quartile 1; Q2, quartile 2; Q3, quartile 3; Q4, quartile 4

## Discussion

In our population-based study, we observed an association of the TyG index with the risk of 1-year MACEs in patients with CHD and hypertension. Consequently, this study suggested that within a certain range, a higher TyG index indicated a higher incidence of MACEs in a population of patients with both CHD and hypertension among one year. RCS model based on logistic regression models indicated that the TyG index had a nonlinear relationship with the probability of the risk of 1-year MACEs. After adjusting for confounding variables, the TyG index was still significantly related to increasing risk of MACEs, and the highest TyG index values enhanced the risk by 47.0% over the 1-year follow-up.

Recently, the TyG index has been widely established as a simple and reliable surrogate marker for IR and has proved to be independently linked with the incidence of diabetes [18, 19]. Previous studies have confirmed that an elevated level of the TyG index was closely related to a higher risk of cardiovascular events and death [20, 21]. After a median follow-up of 98.2 months [22], it was found that the TyG index carried a 0.1- and 0.29-fold elevated risk of all-cause and cardiovascular death in the general population, respectively. According to a retrospective observational study [23], the TyG index was a strong predictor of coronary and carotid atherosclerosis in patients with symptomatic CHD, which is of higher value than FBG or TG levels alone in predicting diseases. Another study [24] involving 4,600 participants showed that a high TyG index was significantly associated with an increased risk of new-onset hypertension in Chinese adults, and maintaining a relatively low TyG index level might be beneficial for primary prevention of hypertension. In addition to hypertension-mediated organ damage, it was reported that the TyG index was positively associated with albuminuria among hypertensive participants [25]. Nevertheless, there are few data to support relationships between the TyG index and 1-year risk of MACEs from both CHD and hypertension.

As a convenient and easy-to-obtain measure of IR, TyG index can not only predict the occurrence of diabetes [26], hypertension [27, 28], atherosclerotic cardiovascular disease [29–31] and even tumor-related diseases [32, 33], but also can be used as a clinical prognostic indicator of some CVDs [21, 34, 35]. Hypertension and diabetes are the most important risk factors for CVDs and play a crucial role in the occurrence and development of CHD [36]. This means that patients with CHD with hypertension or diabetes have a worse cardiovascular prognosis, which has been confirmed in previous studies [37]. Furthermore, there is accumulating evidence suggesting that elevated TyG index is associated with adverse outcomes in CHD patient [9]. However, the TyG index for the prognostic value for patients with CHD and hypertension remains poorly known. In the present study, after the multivariate regression analysis and the subgroup analysis, we found that the TyG index had independent relevance to the risk of 1-year MACEs among patients with both CHD and hypertension. Besides, our findings also revealed that individuals who experienced MACEs had higher TyG index values over one year compared to people who did not experience adverse events in patients with CHD and hypertension, and this trend was even more pronounced in people with diabetes. Subgroup analysis also suggested that elderly patients (≥ 65 years) had a significant role in the association between TyG index and 1-year MACEs compared to those younger than 65 years. Theoretically, it has been reported that aging could be related to poor glucose tolerance because insulin secretion decreases with age [38]. Taking the TyG index as a simple method of evaluating the extent of IR, our findings indicated that IR was independently related to the risk of MACEs with one year. Some studies have reported the pathogenesis behind this effect between the TyG index and MACEs, which may be explained by the severity of the disease reflected in IR [39, 40]. IR, characterized by a significant decline in glucose metabolism in response to insulin, is thought to be a contributory factor of chronic hyperglycemia, dyslipidemia, and hypertension [41]. Further oxidative stress and inflammatory responses lead to endothelial dysfunction and cell damage [42, 43].

Our study innovatively found that patients with both CHD and hypertension in the MACEs group had altered cardiac structure and function at baseline, mainly with significantly higher LAD, LVDd, IVST, PWT, and lower LVEF than those in the non-MACEs group. Similarly, patients with poor peripheral artery status were more likely to have adverse cardiovascular events in individuals with CHD and hypertension, which were manifested by higher baPWV, lower FMD and lower ABI values. Moreover, 379 (46.79%) suffered from MACEs within one year out of 810 patients and the incidence of MACEs was higher in our study than in previous studies. One reason for this result may be that a compound cardiovascular outcome of the first occurrence of MACEs within one-year follow-up, including cardiac death, unplanned revascularization, in-stent restenosis, non-fatal myocardial infarction, stroke, and unplanned rehospitalization for cardiac causes, was used as the endpoint of the clinical trial. Besides, inclusion of single-center patients may lead to selection bias and the external validity of our results need to be further examined. In summary, the novel finding of this study was that the cardiac function and structure and peripheral artery function were important factors influencing cardiovascular prognosis. Another study [44] examining the relationship between T-wave abnormalities and adverse cardiovascular events and echocardiographic changes in hypertensive patients also produced similar findings. In the RCS model and subgroup analysis, our study found that the nonlinear relationship between the TyG index and 1-year MACEs in patients with CHD and hypertension and had an interaction with gender (*P* for interaction < 0.05). This study showed that there was no significant association of the TyG index with 1-year MACEs in male and female. Differently, gender differences in IR-related CVDs risk have previously been reported [45, 46].

The mechanism of TyG index associated with CVDs and adverse cardiovascular outcomes has not been clearly illustrated. TyG is an indicator composed of two risk factors for CVDs, both lipid-related and glucose-related factors reflect IR in the adults, which may be one of the explanations for this association [47–49]. In the first place, IR-induced imbalance of glucose metabolism and lipid metabolism could cause inflammation and oxidative stress, leading to the occurrence and development of atherosclerosis [50, 51]. Second, impaired release and reduced bioavailability of nitric oxide associated with IR can damage the vascular endothelium and lead to inflammation, endothelium-dependent vasodilation and hypertension [52, 53]. Besides, IR is also closely linked with the excessive production of reactive oxygen stress, which can lead to endothelial function impairment [54]. Third, IR may cause platelet overactivity, abnormal platelet adhesion induction, and increased expression of thromboxane A2-dependent tissue factors, ultimately leading to thrombosis and inflammation [55]. Finally, IR with hyperglycemia will induce excessive glycosylation, thereby facilitating smooth muscle cell proliferation, collagen cross-linking and collagen deposition, which is closely associated with substantial increases in the prevalence of vascular fibrosis and stiffness. Those pathologic change will lead to cardiac fibrosis, increased diastolic left ventricular stiffness, and eventually result in cardiac structural and functional abnormalities [52, 56, 57]. Moreover, other indicators such as the atherogenic index of plasma (AIP), a logarithmic conversion of TG to HDL-C molar concentrations, also has been considered to be crucial in predicting and diagnosing CVDs [58].

Several limitations of this trial should be considered. Firstly, conducting a study at a single center may impact its external validity. Secondly, the study’s sample size was modest, which reduced the statistical power and increased the chance of type 2 errors. Thirdly, laboratory parameters were only detected once on admission, and changes during the one-year follow-up period may cause deviations in the analysis results. To overcome these limitations, future studies should aim to capture comprehensive clinical information and track changes in TyG index values over time and focus on multicenter, more rigorous studies with larger sample sizes and extended follow-up periods to provide more robust evidence to corroborate our findings.

## Conclusion

As a result, our study shows that TyG is a potential predictor of 1-year MACEs in patients with CHD and hypertension, and this relationship remained significant after adjustment for other confounders. In this high-risk group, TyG might be a valuable tool for risk categorization and management. Therefore, in clinical work, for patients with CHD and hypertension, TyG index should be paid attention and closely monitored while strengthening the control of traditional cardiovascular risk factors including hypertension. Additionally, further research is required to confirm these results and identify the mechanisms behind the link between TyG and prognosis in patients with CHD and hypertension.

### Electronic supplementary material

Below is the link to the electronic supplementary material.

Supplementary Material 1

## Data Availability

The datasets are not publicly available because the individual privacy of the participants should be protected. Data are however available from the corresponding author on reasonable request.

## Abbreviations

TyG: Triglyceride-glucose
CHD: Coronary heart disease
MACE: Major adverse cardiovascular event
CVD: Cardiovascular disease
IR: Insulin resistance
BMI: Body mass index
SBP: Systolic blood pressure
DBP: Diastolic blood pressure
HR: Heart rate
CKD: Chronic kidney disease
OMI: Old myocardial infarction
ACEI: Angiotensin converting enzyme inhibitor
ARB: Angiotensin receptor blocker
CCB: Calcium channel blockers
TC: Total cholesterol
TG: Triglyceride
LDL-C: Low-density lipoprotein cholesterol
HDL-C: High-density lipoprotein cholesterol
HCY: Homocysteine
Hs-CRP: Hypersensitive C-reactive protein
Scr: Serum creatinine
FBG: Fasting blood glucose
HbA1c: Glycosylated hemoglobin
PAD: Peripheral artery disease
baPWV: Brachial-ankle pulse wave velocity
ABI: Ankle-brachial index
FMD: Brachial artery flow-mediated vasodilatation
LAD: Left atrial diameter
LVDd: Left ventricular end-diastolic diameter
IVST: Interventricular septal thickness
PWT: Left ventricular posterior wall thickness
LVEF: Left ventricular ejection fraction
RCS: Restricted cubic spline
AIP: Atherogenic index of plasma
