# Supplementary material for: Association between the triglyceride-glucose index and 1-year major adverse cardiovascular events in patients with coronary heart disease and hypertension
Source: Cardiovasc Diabetol. 2023 Nov 8;22:305. doi: 10.1186/s12933-023-02018-9 (PMC10633928; doi:10.1186/s12933-023-02018-9)
Supplement: Supplementary file 1 — Supplementary Material 1 [file 12933_2023_2018_MOESM1_ESM.pdf]

Additional file 1

Table S1 The main baseline characteristics for eligible patients according to the TyG quartiles

Indicators	Q1≤8.48	8.48<Q2≤8.85	8.85<Q3≤9.18	Q4>9.18	*P* value
N	205	213	191	201	
TyG	8.26 (8.08, 8.4)	8.71 (8.59, 8.78)	9.02 (8.95, 9.1)	9.47 (9.3, 9.74)	<0.001
Male (n, %)	158 (77.07)	127 (59.62)	139 (72.77)	147 (73.13)	0.001
Age (years)	69 (60, 75.5)	67 (56.5, 73)	66 (57, 75)	63 (56, 71)	0.009
BMI (kg/m^2)	24.68 (22.85, 26.42)	24.39 (23.03, 27.55)	25.39 (23.39, 27.47)	25.86 (23.88, 27.98)	<0.001
SBP (mmHg)	134 (122, 148)	133 (120, 146.5)	134 (122, 149)	140 (129, 154.5)	<0.001
DBP (mmHg)	79 (69, 86)	77 (68, 84)	78 (69, 86)	81 (72, 89)	0.001
HR (bpm)	72 (66, 80)	70 (67, 78)	73 (68, 80)	76 (70, 83)	<0.001
Smoking (n, %)	83 (40.49)	80 (37.56)	98 (51.31)	97 (48.26)	0.017
Drinking (n, %)	42 (20.49)	43 (20.19)	55 (28.8)	57 (28.36)	0.058
Case history (n, (%)					
Diabetes	70 (34.15)	102 (47.89)	91 (47.64)	140 (69.65)	<0.001
CKD [a]	25 (12.2)	22 (10.33)	33 (17.28)	34 (16.92)	0.110
OMI	31 (15.12)	28 (13.15)	48 (25.13)	44 (21.89)	0.005
Family history of CVDs	56 (27.32)	65 (30.52)	73 (38.22)	64 (31.84)	0.128
Number of coronary lesions (n, %)					
One-vessel disease	161 (78.54)	172 (80.75)	155 (81.15)	141 (70.15)	0.027
Two-vessel disease	34 (16.59)	36 (16.9)	25 (13.09)	51 (25.37)	0.012
Multi-vessel disease	10 (4.88)	5 (2.35)	11 (5.76)	9 (4.48)	0.375
Cardiovascular medications (n, %)					
Anti-platelet	205 (100)	213 (100)	190 (99.48)	201 (100)	0.236
Statins	204 (99.51)	208 (97.65)	187 (97.91)	197 (98.01)	0.508
ACEI/ARB	139 (67.8)	145 (68.08)	129 (67.54)	139 (69.15)	0.987
β-blockers	150 (73.17)	162 (76.06)	147 (76.96)	154 (76.62)	0.803
CCB	75 (36.59)	92 (43.19)	74 (38.74)	103 (51.24)	0.016
Nitrates	70 (34.15)	76 (35.68)	53 (27.75)	52 (25.87)	0.088
Laboratory variables					
TC (mmol/L)	3.62 (2.86, 4.12)	3.67 (3.18, 4.48)	3.99 (3.25, 5.05)	4.45 (3.63, 5.45)	<0.001
TG (mmol/L)	0.88 (0.77, 1.06)	1.29 (1.12, 1.46)	1.77 (1.52, 1.98)	2.32 (1.87, 3.11)	<0.001
LDL-C (mmol/L)	2.13 (1.61, 2.5)	2.17 (1.83, 2.68)	2.34 (1.84, 3.1)	2.63 (2.07, 3.18)	<0.001
HDL-C (mmol/L)	1.04 (0.88, 1.23)	1 (0.9, 1.17)	0.94 (0.76, 1.1)	0.97 (0.79, 1.11)	<0.001
HCY (μmol/L)	14.18 (11.55, 17.35)	12.6 (10.96, 16.18)	15.03 (11.68, 20.65)	14.18 (11.98, 18.82)	<0.001
Hs-CRP (mg/L)	2.36 (1, 5.7)	2.1 (0.99, 5.7)	3.12 (1.14, 5.7)	2.64 (1.44, 5.7)	0.182
Scr (μmol/L)	79 (67.55, 92.05)	72.4 (59.25, 85.65)	77.2 (64.5, 94.7)	74.3 (61.45, 89.8)	0.001
FBG (mmol/L)	5.2 (4.76, 5.85)	5.82 (5.15, 6.37)	5.92 (5.25, 6.99)	7.1 (6.14, 8.98)	<0.001
HbA1c (%)	5.9 (5.6, 6.3)	6.1 (5.75, 6.8)	6.4 (5.9, 7.2)	7 (5.9, 8.3)	<0.001
PAD indicators					
baPWV (m/s)	16.25 (15.27, 21.29)	16.16 (14.97, 20)	20.55 (16.33, 23.67)	18.92 (15.44, 23.21)	<0.001
ABI	1.12 (0.96, 1.18)	1.14 (1.01, 1.19)	0.99 (0.89, 1.14)	1.01 (0.88, 1.15)	<0.001
FMD (%)	7.1 (6.1, 8.1)	7.1 (6.3, 8.3)	6.3 (5.8, 7.5)	6.5 (5.9, 7.7)	<0.001
Echocardiography					
LAD (mm)	37 (35, 40)	38 (36, 40)	38 (36, 40)	38 (36, 41)	0.032
LVDd (mm)	51 (48, 54)	50 (47, 54)	51.19 (49, 54)	51 (48, 54)	0.029
IVST (mm)	10 (9, 11)	10 (9, 11)	10 (10, 12)	11 (10, 12)	<0.001
PWT (mm)	9 (8, 9.13)	9 (8, 10)	9 (8, 10)	10 (9, 10)	<0.001
LVEF (%)	65 (62, 69)	66 (60, 70.5)	64 (58, 69)	64 (60, 70)	0.366

TyG, triglyceride-glucose; Q1, quartile 1; Q2, quartile 2; Q3, quartile 3; Q4, quartile 4; BMI, body mass index; SBP, systolic blood pressure; DBP, diastolic blood pressure; HR, heart rate; CKD, chronic kidney disease; OMI, old myocardial infarction; CVD, cardiovascular disease; ACEI, angiotensin converting enzyme inhibitor; ARB, angiotensin receptor blocker; CCB, calcium channel blockers; TC, total cholesterol; TG, triglyceride; LDL-C, low-density lipoprotein cholesterol; HDL-C, high-density lipoprotein cholesterol; HCY, homocysteine; Hs-CRP, hypersensitive C-reactive protein; Scr, serum creatinine; FBG, fasting blood glucose; HbA1c, glycosylated hemoglobin; PAD, peripheral artery disease; baPWV, brachial-ankle pulse wave velocity; ABI, ankle-brachial index; FMD,

brachial artery flow-mediated vasodilatation; LAD, left atrial diameter; LVDd, left ventricular end-diastolic diameter; IVST, interventricular septal thickness; PWT, left ventricular posterior wall thickness; LVEF, left ventricular ejection fraction
[a] Defined as eGFR < 60 ml/min/1.73 m^2 on the basis of The KDIGO CKD Clinical Guideline

Table S2 Collinearity diagnostics among the variables

Indicators	VIF
Gender	1.878
Age	1.785
BMI	1.349
SBP	1.667
DBP	1.910
HR	1.328
Smoking	1.901
Drinking	1.532
Case history	
Diabetes	1.669
CKD [a]	1.465
OMI	1.263
Family history of CVDs	1.145
Number of coronary lesions	1.104
Cardiovascular medications	
Anti-platelet	1.063
Statins	1.035
ACEI/ARB	1.137
β-blockers	1.113
CCB	1.177
Nitrates	1.114
Laboratory variables	
TC	1.356
TG	5.685
LDL-C	1.510
HDL-C	1.081
HCY	1.394
Hs-CRP	1.123
Scr	1.427
FBG	3.292
HbA1c	2.331
TyG	7.905
PAD indicators	
baPWV	2.636
ABI	2.591
FMD	1.613
Echocardiography	
LAD	1.675
LVDd	2.186
IVST	1.987
PWT	1.896
LVEF	1.828

VIF, variance inflation factor; TyG, triglyceride-glucose; BMI, body mass index; SBP, systolic blood pressure; DBP, diastolic blood pressure; HR, heart rate; CKD, chronic kidney disease; OMI, old myocardial infarction; CVD, cardiovascular disease; ACEI, angiotensin converting enzyme inhibitor; ARB, angiotensin receptor blocker; CCB, calcium channel blockers; TC, total cholesterol; TG, triglyceride; LDL-C, low-density lipoprotein cholesterol; HDL-C, high-density lipoprotein cholesterol; HCY, homocysteine; Hs-CRP, hypersensitive C-reactive protein; Scr, serum creatinine; FBG, fasting blood glucose; HbA1c, glycosylated hemoglobin; PAD, peripheral artery disease; baPWV, brachial-ankle pulse wave velocity; ABI, ankle-brachial index; FMD, brachial artery flow-mediated vasodilatation; LAD, left atrial diameter; LVDd, left ventricular end-diastolic diameter; IVST, interventricular septal thickness; PWT, left ventricular posterior wall thickness; LVEF, left ventricular ejection fraction
[a] Defined as eGFR < 60 ml/min/1.73 m^2 on the basis of The KDIGO CKD Clinical Guideline
No potentially significant collinearity is defined as VIF less than 10 among variables
